# IL-17 Induced Stromal Cell–Derived Factor-1 and Profibrotic Factor in Keloid-Derived Skin Fibroblasts *via* the STAT3 Pathway

**DOI:** 10.1007/s10753-019-01148-1

**Published:** 2019-12-09

**Authors:** Seon-Yeong Lee, Eun Kyung Kim, Hyun Beom Seo, Jeong Won Choi, Jin Hee Yoo, Kyoung Ah Jung, Da-Som Kim, Seung Cheon Yang, Soo Jin Moon, Jung Ho Lee, Mi-La Cho

**Affiliations:** 1 The Rheumatism Research Center, Catholic Research Institute of Medical Science, College of Medicine, The Catholic University of Korea, Seoul, Republic of Korea; 2 Laboratory of Immune Network, Conversant Research Consortium in Immunologic Disease, College of Medicine, The Catholic University of Korea, 222, Banpo-daero, Seocho-gu, Seoul, 06591 Republic of Korea; 3 Department of Internal Medicine, College of Medicine, Division for Rheumatology, The Catholic University of Korea, Seoul, Republic of Korea; 4 Department of Plastic and Reconstructive Surgery, Bucheon St. Mary’s Hospital, College of Medicine, The Catholic University of Korea, 2 Sosa-dong, Wonmi-gu, Bucheon-si, Gyeonggi-do 420-717 Republic of Korea

**Keywords:** keloid, fibroblast, IL-17, SDF-1, STAT3

## Abstract

The pathogenesis of keloids has not been elucidated, and the disease is thought to be caused by abnormal secretion of proinflammatory mediators and irregular responses to other inflammatory signals mediated by keloid fibroblasts (KFs). In this study, we investigated whether a local increase in interleukin IL-17 in keloid tissues stimulates the production of stromal cell–derived factor-1 (SDF-1) in KFs causing further recruitment of IL-17-producing T helper 17 (Th17) cells, which subsequently creates a positive feedback loop. Histological assessment was performed and the change in the expression of IL-17, IL-1β, IL-6, and TNF-α which of fibrosis and inflammation associated markers was examined. In addition, fibroblasts were treated with IL-17 in the presence or absence of STAT3 inhibitor STA-21; SDF-1 levels and fibrosis genes were measured. Our results showed that fibrotic reaction and expression of proinflammatory cytokines including IL-17 were most prominent in the growing margin (perilesional area) of keloid tissue and Th17 cells significantly infiltrated the perilesional area. In addition, IL-17 upregulated the expression of SDF-1, collagen, and α-SMA in KFs. Finally, STA-21 decreased SDF-1α expression and the expression of fibrosis genes in KFs even after IL-17 stimulation. Our study demonstrated that a local increase in IL-17 in keloid tissues stimulates the production of SDF-1 in KFs causing further recruitment of IL-17-producing T helper 17 (Th17) cells, which subsequently creates a positive feedback loop. These findings suggest that STAT3 inhibition can be used to treat keloid scars by reversing the vicious cycle between Th17 cells and KFs.

## INTRODUCTION

Pathologic fibroproliferative disorders such as keloids and hypertrophic scars are characterized by excessive collagen deposition and the formation of raised dermal lesions. The pathogenesis of keloids has not been elucidated, although both environmental and genetic risk factors have been implicated. Previous studies have shown the presence of chronic inflammation in keloids, suggesting the disease is caused by the abnormal secretion of proinflammatory mediators and irregular responses to other inflammatory signals mediated by KFs [1, 2]. However, the pathogenesis of keloids and tissue fibrosis has not been elucidated and exact molecular are still unclear.

Many studies have shown the profibrotic effects of interleukin (IL)-17 [3, 4]. Recombinant IL-17A increased the production of collagen from mouse skin fibroblasts in a dose-dependent manner [5]. In addition, IL-17A increased profibrotic cytokines such as TGF-β and connective tissue growth factor in skin fibroblasts [6]. In another study, recombinant IL-17A enhanced proliferation of pulmonary fibroblasts and increased the production of collagen, TGF-β, and IL-6, which was mitigated by anti-IL-17A treatment [3]. Recently, Zhang et al. found that elevated IL-6 and TGF-β in keloid tissue promoted the differentiation of naïve T (Th0) cells into IL-17^+^ T helper (Th) 17 cells and further stimulated IL-6 secretion *via* IL-17, thus creating an enriched proinflammatory cytokine milieu [7]. However, the exact role of Th17 cells in the formation of keloid tissue remains unknown. In a previous study, we demonstrated that IL-17 in T cells stimulates fibroblast-like synoviocytes (FLSs) to produce SDF-1 in a dose-dependent manner, indicating a reciprocal action between T cells and FLSs in the pathogenesis of rheumatoid arthritis (RA) [8]. In other words, in patients with RA, T cells migrate into the synovium guided by SDF-1 from FLSs, and IL-17 in recruited T cells increases the production of SDF-1 in FLSs, resulting in augmentation of the inflammatory process.

In this study, we have investigated whether a similar mechanism exists in the formation of keloid tissue. A local increase in IL-17 in keloid tissue may stimulate production of SDF-1 in KFs and further enhance the recruitment of Th17 cells from the bloodstream, resulting in the formation of a positive feedback loop. We assessed whether infiltration of Th17 cells is increased in keloid tissue, and whether paracrine signaling from KFs (SDF-1) exerts chemotactic effects on Th17 cells. In addition, we have investigated the effects of IL-17 on SDF-1 produced by KFs and the profibrotic effects of IL-17 on KFs, as well as determined the inhibitory effects of STA-21 on SDF-1α produced by KFs.

## MATERIALS AND METHODS

### Isolation and Culture of Human KFs

Fibroblasts were isolated by enzymatic digestion of keloid tissue specimens obtained from 6 patients with keloids undergoing scar revision surgery. The tissue samples were minced into 2- to 3-mm pieces and treated for 30 min with 2.5 mg/mL type I collagenase (Sigma-Aldrich, St. Louis, MO, USA) in phosphate-buffered saline (PBS) at 37 °C in 5% CO_2_. Dissociated cells were centrifuged at 1300 rpm, resuspended in Dulbecco’s modified Eagle’s medium (DMEM) supplemented with 10% fetal bovine serum (FBS), 2 mM l-glutamine, 100 units/mL penicillin, and 100 ng/mL streptomycin, and plated in 25-cm^2^ flasks. After overnight culture, floating cells were removed, and the adherent cells were cultivated in DMEM supplemented with 10% FBS. The cultures were kept at 37 °C in 5% CO_2_, and the medium was replaced every 3 days. The fibroblasts were passaged 3–8 times using trypsin–ethylenediaminetetraacetic acid (EDTA) (Gibco, Grand Island, NY, USA). The cells were seeded in 24-well plates in DMEM supplemented with 10% FBS and then cultured for 48 h at 37 °C. Fibroblasts were stained with allophycocyanin-conjugated anti-CD90 (eBioscience, San Diego, CA, USA) antibody as a fibroblast marker and analyzed by flow cytometry. Informed consent was obtained from all participating subjects. The study received approval from the Institutional Review Board for Human Research of Bucheon St. Mary’s Hospital (HIRB-20180322-001).

### Histological Assessment

Human keloid tissue samples of 6 patients were fixed in 10% neutral buffered formalin and embedded in paraffin. The tissues were sectioned at a thickness of 6 μm, deparaffined using xylene, dehydrated in a graded alcohol series, and then stained with hematoxylin and eosin or Masson’s trichrome. Immunohistochemical staining was performed using the Vectastain ABC kit (Vector Laboratories, Burlingame, CA, USA). The tissues were incubated with anti-IL-17, anti-IL-1β, anti-IL-6, and anti-TNF-α (Abcam, Cambridge, MA, USA) overnight at 4 °C. The primary antibodies were detected using a biotinylated secondary antibody for 40 min, followed by incubation with a streptavidin–peroxidase complex for 1 h. The final color product was developed using 3, 3′-diaminobenzidine chromogen (DAKO, Carpinteria, CA, USA). Positive cells were counted and the numbers expressed as means ± standard deviation.

### Confocal Microscopy

For immunostaining, 5-μm-thick keloid tissue sections were fixed and permeabilized with acetone, washed with PBS, and then blocked with 10% normal goat serum for 30 min. To analyze the populations of T helper cells and STAT, the tissues were stained with fluorescein isothiocyanate (FITC)-conjugated anti-CD4 (BD Biosciences, Sparks, MD, USA), phycoerythrin (PE)-conjugated anti-IL-17 (BioLegend, San Diego, CA, USA), or/and PE-conjugated anti-phosphorylated STAT3 tyrosine 705 and PE-conjugated anti-phosphorylated STAT3 tyrosine 727 (BD Biosciences) antibodies. For fibroblast analysis, the tissues were stained with PE-conjugated anti-CD90 (eBioscience) and FITC-conjugated anti-SDF-1 (R&D Systems, Minneapolis, MN, USA) antibodies overnight at 4 °C. The primary antibody was detected using a FITC-conjugated anti-rabbit IgG secondary antibody for 2 h at room temperature, and the nuclei were stained with 4′,6-diamidino-2-phenylindole. The stained tissues were analyzed using a Zeiss microscope (LSM 510 Meta; Carl Zeiss, Oberkochen, Germany) at × 400 magnification.

### Isolation of CD4^+^ T Cells and Transwell Migration Assay

To purify human CD4^+^ T cells, peripheral blood mononuclear cells were incubated with CD4-coated magnetic beads and isolated using magnetic-activated cell sorting separation columns (Miltenyi Biotec, Bergisch Gladbach, Germany). For Th0 cell-polarizing, the isolated CD4^+^ T cells were stimulated with plate-bound anti-CD3 (0.5 μg/mL), anti-CD28 (1 μg/mL) to Th0 polarizing. For Th17 cell-polarizing, the isolated T cells were cultured with plate-bound anti-CD3 (0.5 μg/mL), anti-CD28 (1 μg/mL), anti-IFN-g (2 μg/mL), anti-IL-4 (2 μg/mL), anti-IL-2 (2 μg/mL), TGF-β (2 ng/mL), IL-23 (20 ng/mL), and IL-6 (20 ng/mL) for 72 h. Migration assays were performed in a 24-well Transwell unit with a 3-μm-pore size (Corning Costar, Cambridge, MA, USA). 2 × 10^4^ KFs were seeded in the lower chamber for 1 day before culture with Th17 cells. After 1 day, 2 × 10^5^ Th17 cells were seeded in the upper chamber of the Transwell assembly. The lower chamber contained 600 μL medium supplemented with SDF-1α (10 ng/mL; PeproTech, Rocky Hill, NJ, USA). The cells were cultured for 4 h, and the number of Th17 cells that migrated toward the keloid cells was counted.

### Gene Expression Analysis Using Quantitative Real-Time Polymerase Chain Reaction

Total RNA was extracted using TRIzol (Molecular Research Center, Cincinnati, OH, USA), and 2 μg total RNA were reverse transcribed using the Superscript Reverse Transcription system (Takara, Shiga, Japan). Quantitative real-time polymerase chain reaction (qRT-PCR) was performed using Light-Cycler FastStart DNA master SYBR green I (Takara) fluorescent dye on the ABI PCR system. Primers targeting TGF-β (forward: 5′- TGC GGC AGC TGT ACA TTG A -3′, reverse: 5′- TGG TTG TAC AGG GCC AGG A -3′), α-SMA (forward: 5′- TGG GTG ACG AAG CAC AGA GC -3′, reverse: 5′- CTT CAG GGG CAA CAC GAA GC -3′), collagen-1 (forward: 5′- GTC ACC CAC CGA CCA AGA AAC C -3′, reverse: 5′- AAG TCC AGG CTG TCC AGG GAT G -3′), and β-actin (forward: 5′- GGA CTT CGA GCA AGA GAT GG -3′, reverse: 5′- TGT GTT GGG GTA CAG GTC TTT G -3′) were designed using Primer Express (Applied Biosystems, Foster City, CA, USA). The mRNA expression level of each gene was normalized to that of β-actin.

### Enzyme-Linked Immunosorbent Assay

2 × 10^4^ KFs were cultured in 0.1% ITSA-DMEM in 24-well plates in the presence or absence of IL-17 (5, 20, 50 ng/mL), and STA-21 (10 μM) for 48 h. Human-derived SDF-1 was measured in the culture supernatants by sandwich (enzyme-linked immunosorbent assay) ELISA (R&D Systems). Alkaline phosphatase (Sigma-Aldrich) was used for color development. Absorbance was measured at 405 nm on an ELISA microplate reader (Molecular Devices, Sunnyvale, CA, USA).

### Statistical Analyses

Statistical analyses were preformed using GraphPad Prism 5 software. Significant differences between treatment groups were assessed using the Mann–Whitney *U* test. The results are expressed as means ± standard deviation or means ± standard error of the mean. *P* < 0.05 (two-tailed) was considered to indicate statistical significance.

## Results

### Increased Expression of Fibrosis Markers Results in Keloid Scar Formation

**Fig. 1:**
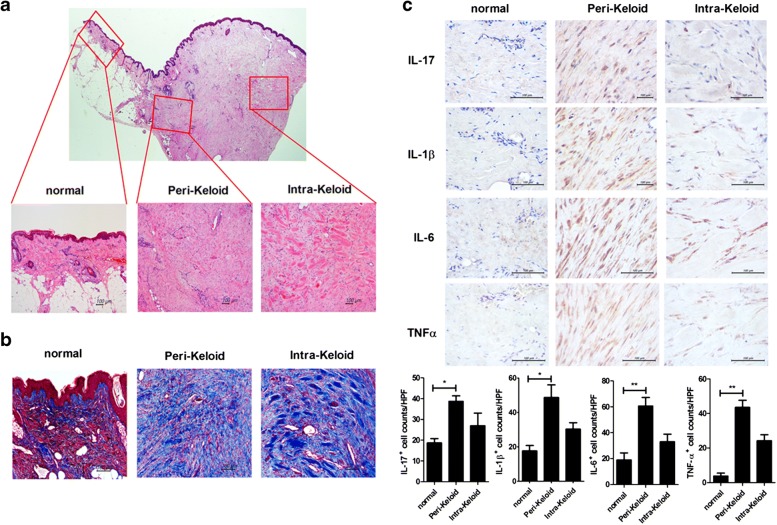
Different degrees of inflammation and fibrosis according to the location. Human keloid tissues were obtained of each of six patients. **a**, **b** The human keloid tissues were stained with hematoxylin and eosin (H&E) or Masson’s trichrome (MT) staining. The inflammatory reaction in keloid tissue is most prominent in the perilesional area. **c** Immunohistochemical staining for IL-17, IL-1β, IL-6, and TNF-α according to the location. Degress of infiltration of IL-17-positive cells and IL-6-positives cells according to the location. Original magnification × 100, × 200. Data represent the mean ± SD of six independent experiments (*P < 0.05; **P < 0.01).

To investigate fibrosis degree during keloid development, the keloid tissue was stained with hematoxylin and eosin and Masson’s trichrome. Histological analyses showed that infiltration of lymphocytes was increased in the perilesional area (growing margin of keloids; Fig. 1a) compared with normal tissue area. In addition, fibrosis was observed more frequently increased in the perilesional keloid tissues than intralesional keloid tissues, indicating that keloid scars are benign fibroproliferative dermal tumors (Fig. 1b) [9].

### The Pathogenesis of Keloids Is Closely Related to Infiltration of Proinflammatory Cytokines

Next, we assessed whether the keloid scar lesion accompanied by fibrosis increases the expression of proinflammatory cytokines. Immunohistochemical staining showed IL-17, IL-1β, IL-6, and TNF-α level were increased in the perilesional area of keloids compared with normal tissue or the keloid intralesional area (Fig. 1c).

### Activation of IL-17 and SDF-1 in the Perilesional Area of Keloids

**Fig. 2:**
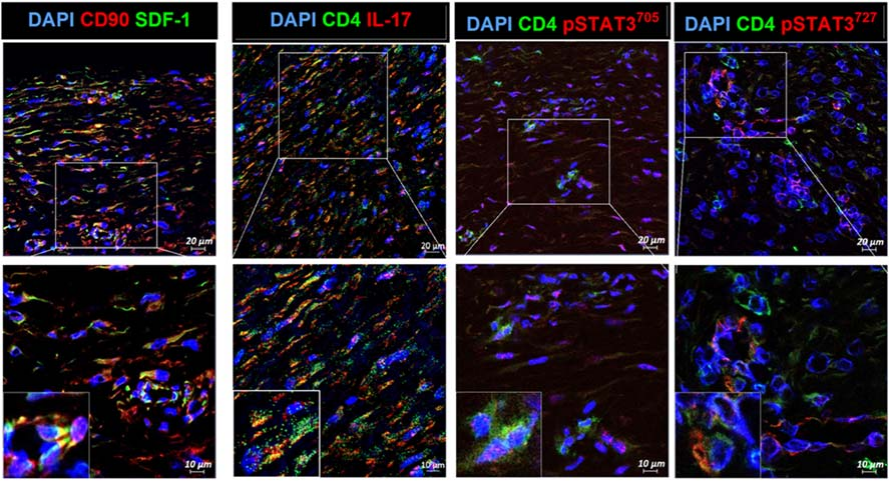
Confocal microscopy for the perilesional area of keloids. Human keloid tissues were obtained of each of six patients and were repeated 3 times. Increased SDF-1 expression from KFs and increased infiltration of Th17 are shown. Original magnification × 200 and × 400.

Confocal microscopy showed colocalization of CD90^+^SDF-1^+^ and CD4^+^IL-17^+^ cells in the same region of the perilesional area. In addition, IL-17-producing CD4^+^pSTAT3 705^+^ and CD4^+^pSTAT3 727^+^ cells were increased in abundance in the perilesional area of keloids. These data indicate that increased SDF-1 expression in keloid tissue can stimulate the infiltration of Th17 cells (Fig. 2).

### The Role of KFs to Th17 Cell Migration

**Fig. 3:**
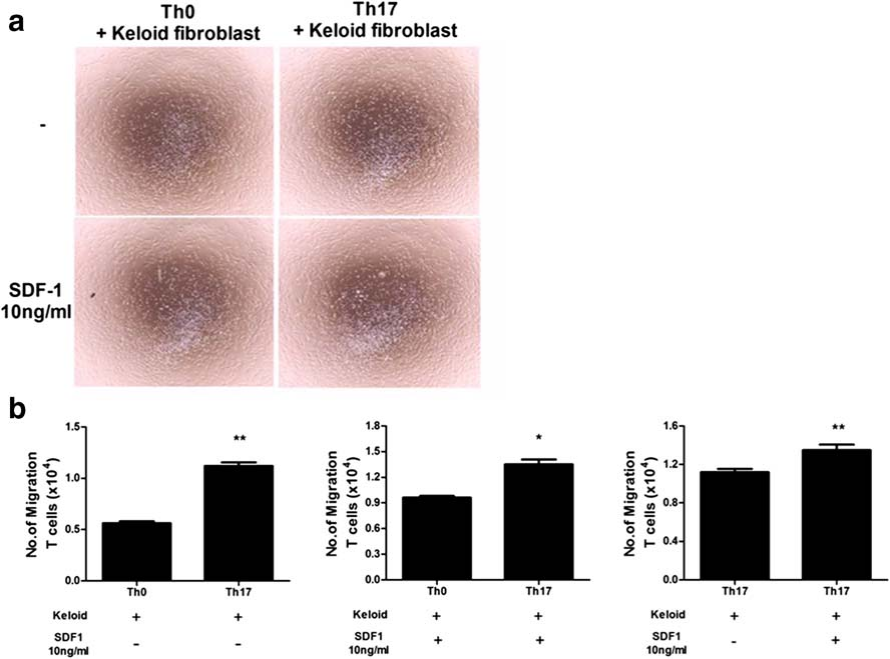
Transwell migration assay. Compared with Th0 cells, the migration of Th17 cells was significantly increased in the presence or absence of SDF. **a** Optical microscopic images of the migrating cells. Original magnification × 40. **b** The number of migrated T cells. Data represent the mean ± SD of three independent experiments (*P < 0.05; **P < 0.01).

To further investigate the interaction between Th17 cells and KFs, we evaluated the migration of naive T (Th0) cells Th17 cells co-cultured with KFs in the presence or absence of SDF-1. Compared with Th0 cells, the migration of Th17 cells was significantly increased in the presence or absence of SDF. In addition, the migration of Th17 cells was significantly increased in the presence of SDF-1 compared with in the absence of it (Fig. 3). These data indicate that IL-17-producing T cells have a high migration capacity toward KFs and SDF-1 augments it.

### IL-17 Stimulates the Expression of SDF-1 and Fibrosis Markers in KFs

**Fig. 4:**
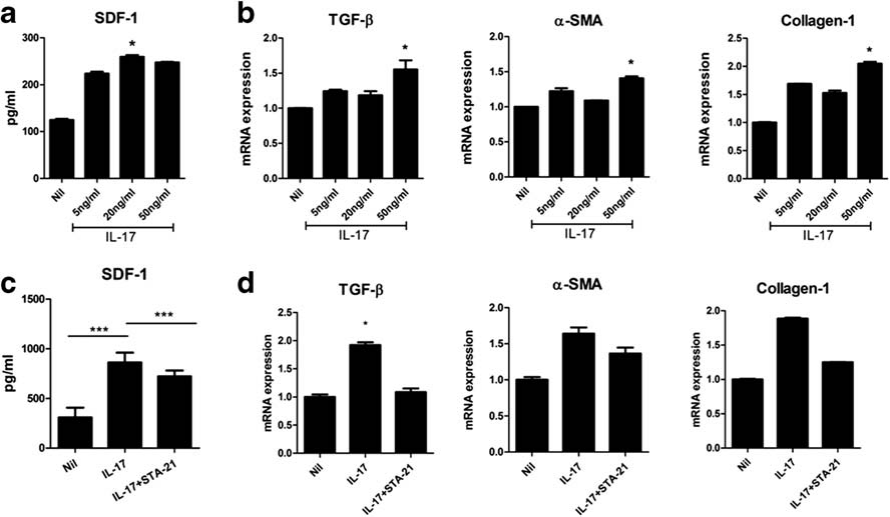
Increased expression of SDF-1 and profibrotic markers in KFs under the influence of IL-17 in KFs. **a** SDF-1 levels in culture supernatants were measured by ELISA. **b** Expression of TGF-β, α-SMA, and collagen were measured by quantitative real-time polymerase chain reaction (qRT-PCR); the results were normalized to β-actin mRNA levels. **c** 2 × 10^4^ KFs were cultured with IL-17 10 ng/mL and STA21 10 μM for 48 h. SDF-1 was measured in culture supernatant using ELISA. **d** Relative mRNA levels of TGF-β, α-SMA, and collagen. Data represent the mean ± SD of three independent experiments (*P < 0.05; **P < 0.01; ***P < 0.001).

KFs were stimulated with IL-17, and the SDF-1 level was measured in the culture supernatants. In addition, the expression of profibrotic genes such as TGF-β, α-SMA, and collagen-1 from KFs was measured under the influence of IL-17. As a result, we found that IL-17 significantly increased production of SDF-1 from KFs and the expression of profibrotic genes was also increased under the influence of IL-17 (Fig. 4a, b). When considering the results of migration assay, these results suggest that a local increase in IL-17 in keloid tissue may stimulate production of SDF-1 in KFs and further enhance the recruitment of Th17 cells, resulting in the formation of a positive feedback loop. Also, this IL-17-rich inflammatory milieu in the keloid tissue could contribute to the excessive fibrosis in it.

### Regulation of SDF-1 and Fibrosis Markers *via* STAT3 Inhibition

The STAT3 transcription factor upregulates IL-17 expression *via* Th17 cell proliferation [10]. Based on the result, we have investigated whether the inflammatory response in KFs could be mediated by the modulation of STAT3 activity using STA-21, a STAT3 inhibitor. KFs were treated with IL-17 and the expression of SDF-1 and profibrotic genes were evaluated in the presence or absence of STA-21. As a result, we found that the increased expression of SDF-1 and profibrotic genes under the influence of IL-17 was significantly decreased in the presence of STA-21 (Fig. 4c, d). This finding demonstrated that the STAT3 inhibition can be used to treat keloid scars by reversing the vicious cycle between Th17 cells and KFs.

## DISCUSSION

The main histopathological characteristics of keloids include abnormal proliferation of fibroblasts and overaccumulation of extracellular matrix components such as collagen [2]. The center of the keloid lesion is highly collagenized and mostly devoid of blood vessels and cells. However, in the perilesional area (growing margin of keloids), numerous lymphocytes and fibroblasts are dispersed among a rich network of blood vessels, suggesting chronic inflammation.

In our study, the expression of proinflammatory cytokines (IL-17, IL-1β, IL-6, and TNF-α) was significantly higher in the perilesional tissue compared with normal tissue or intralesional area. This finding is in accordance with a previous study showing that the inflammatory milieu in keloid tissue promotes resident stem cells to acquire tumor-like cell phenotypes such as increased proliferation and self-renewal [7, 11]. Therefore, to treat keloids and prevent recurrence, modification of the inflammatory niche and reversing the inflammatory process are important.

In this study, we found the increased infiltration of Th17 cells into keloid tissue and hypothesized that excessive recruitment of Th17 cells contribute to the excessive fibrosis in keloid tissue.

In a previous study, we have demonstrated that SDF-1 is overproduced in RA FLSs, and that IL-17 can upregulate SDF-1 expression in RA FLSs *via* the phosphatidylinositol 3-kinase (PI3K) pathway [8]. In addition, Shin et al. showed high infiltration of SDF-1α^+^ myofibroblasts into perilesional keloid tissue and high recruitment of CXCR4^+^ immune cells and CXCR4^+^ fibrocytes in keloids [12]. Based on these results, we speculate that a similar vicious cycle contributes to the chronic inflammation in keloid tissue. In other words, local inflammation from trauma or surgery may increase SDF-1 expression and stimulate recruitment of Th17 cells, potentially resulting in local increases in IL-17 levels and SDF-1 expression in KFs (positive feedback loop).

In the present study, paracrine signaling from KFs stimulated the migration of Th17 cells, which was significantly increased by SDF-1 treatment. In addition, we showed that SDF-1 production in KFs was significantly increased by IL-17 treatment. These findings indicate that SDF-1 in KFs could enhance the recruitment of Th17 cells, and the resulting infiltration of Th17 cells augment SDF-1 expression in KFs, further recruiting more Th17 cells. In addition, we found IL-17 stimulated the expression of profibrotic markers from KFs, indicating that excessive Th17 cell infiltration results in pathological scar formation. This result is in agreement with an earlier study demonstrating that boosting T cell function cells in athymic mice significantly increased the growth of human keloid transplants [9].

Considering these results, suppressing the positive feedback loop between KFs and T cells might be effective in treating pathological scar formation. In other words, the inhibition of SDF-1 expression from KFs can prevent the excessive infiltration of Th-17 cells and the profibrotic effects of IL-17. Our study showed that the STA-21-inhibited STAT3 pathway resulted in decreased production of SDF-1 and fibrosis markers.

In addition to the inhibition the recruitment of effective T cells, decreasing SDF-1 expression may have additional preventive effects on keloid scar formation by impeding fibrocyte homing. Fibrocytes are circulating fibroblast-like mesenchymal progenitor cells that have been associated with fibrotic disorders including pulmonary fibrosis, nephrotic fibrosis, and skin fibrosis [12]. Fibrocytes express surface markers such as CCR2, CCR7, and CCR4, and under inflammatory conditions, they can migrate to the inflammatory site *via* induction by SDF-1α [13]. In keloid and postburn hypertrophic scars, increased infiltration of fibrocytes compared with normal tissue has been reported [12, 14, 15]. Consequently, suppression of SDF-1α expression is expected to decrease fibrotic inflammation as well as the infiltration of fibrocytes.

## CONCLUSIONS

In summary, our study demonstrated that IL-17 enhanced SDF-1 expression in fibroblasts, which in turn increased the migration of Th17 cells from the circulatory system. In addition, IL-17 promoted the expression of profibrotic cytokines including α-SMA and collagen type I in KFs. This positive feedback loop may result in excessive infiltration of T cells and contribute to the chronic inflammation in keloid scars. STA-21 could be used to treat keloid scars by decreasing SDF-1α expression in KFs and breaking the positive feedback loop between KFs and Th17 cells.

## Abbreviations

KF: Keloid fibroblasts
FLS: Fibroblast-like synoviocytes
SDF-1: Stromal cell–derived factor-1
Th17: IL-17-Producing T helper 17 cells
STAT3: Signal transducer and activator of transcription 3
IL: Interleukin
